# Production of d-lactate from glucose using *Klebsiella pneumoniae* mutants

**DOI:** 10.1186/s12934-017-0822-6

**Published:** 2017-11-21

**Authors:** Xinjun Feng, Liqun Jiang, Xiaojuan Han, Xiutao Liu, Zhiqiang Zhao, Huizhou Liu, Mo Xian, Guang Zhao

**Affiliations:** 1 CAS Key Laboratory of Biobased Materials, Qingdao Institute of Bioenergy and Bioprocess Technology, Chinese Academy of Sciences, Qingdao, 266101 China; 2 CAS Key Laboratory of Renewable Energy, Guangzhou Institute of Energy Conversion, Chinese Academy of Sciences, Guangzhou, 510640 China; 3 University of Chinese Academy of Sciences, Beijing, 100049 China

**Keywords:** d-Lactate, Acetolactate synthase, Acetate kinase, Alcohol dehydrogenase, *Klebsiella pneumoniae*

## Abstract

**Background:**

d-Lactate is a valued chemical which can be produced by some bacteria including *Klebsiella pneumoniae*. However, only a few studies have focused on *K. pneumoniae* for d-lactate production with a significant amount of by-products, which complicated the purification process and decreased the yield of d-lactate.

**Results:**

Based on the redirection of carbon towards by-product formation, the effects of single-gene and multiple-gene deletions in *K. pneumoniae* on d-lactate production from glucose via acetolactate synthase (*budB*), acetate kinase (*ackA*), and alcohol dehydrogenase (*adhE*) were tested. *Klebsiella pneumoniae* mutants had different production behaviours. The accumulation of the main by-products was decreased in the mutants. The triple mutant strain had the most powerful ability to produce optically pure d-lactate from glucose, and was tested with xylose and arabinose as carbon sources. Fed-batch fermentation was also carried out under various aeration rates, and the strain accumulated 125.1 g/L d-lactate with a yield of 0.91 g/g glucose at 2.5 vvm.

**Conclusions:**

Knocking out by-product synthesis genes had a remarkable influence on the production and yield of d-lactate. This study demonstrated, for the first time, that *K. pneumoniae* has great potential to convert monosaccharides into d-lactate. The results provide new insights for industrial production of d-lactate by *K. pneumoniae*.

## Background

d-Lactate is an important chiral chemical with widespread applications in herbicides, coatings, adhesive, spices, and cosmetics. d-Lactate is also an excellent monomer for polylactate (PLA) production, a biodegradable plastic. The properties of PLA depend on the monomer composition, and different monomers can be polymerized into different bioplastics, such as poly l-lactate (PLLA), poly d-lactate (PDLA), and poly d, l-lactate (PDLLA). The lower melting point of the traditional homopolymer PLLA restricts its potential applications. Stereocomplex PLA (PDLLA, or sc-PLA), prepared by blending PLLA and PDLA at different ratios, has a melting point of 230 °C, which is 50 °C higher than that of homopolymers; the mechanical performance and hydrolysis resistance of sc-PLA are also improved [1, 2]. With the continued growth of the PLA global market, there will be a great demand for polymer-grade d-lactate.

Biosynthesis of chemicals has attracted great attention because of its high efficiency, sustainable development, and ability to alleviate dependence on petroleum-based materials. d-Lactate can be produced by fermentation using wild-type microbes, such as *Lactobacillus* [3], *Sporolactobacillus* [4] and metabolic engineered strains, including those of the *Escherichia* [5], *Saccharomyces* [6], and *Klebsiella* [7] genera. Blocking the by-products synthesized during metabolic engineering is a primary solution to improve d-lactate production. For example, inactivation of the l-lactate dehydrogenase gene *ldhL1* and phosphoketolase genes *xpK1* and *xpK2* significantly increases d-lactate production [8]. Single-gene deletions of acetate kinase (*ackA*), phosphoenolpyruvate synthase (*pps*), pyruvate formate lyase (*pflB*), FAD-binding d-lactate dehydrogenase (*dld*), pyruvate oxidase (*poxB*), alcohol dehydrogenase (*adhE*), and fumarate reductase (*frdA*) in *Escherichia coli* improved the d-lactate yield. After all seven genes were deleted, the resultant strain generated 125 g/L d-lactate in a 7-L bioreactor [9].

*Klebsiella pneumoniae* is a well-studied Gram-negative bacteria that has a high growth rate in minimal medium and is already widely used as a microbial factory for the production of 3-hydroxypropionate [10] and 1,3-propanediol (1,3-PDO) [11]. It also has a wide variety of substrates, including glycerol and monosaccharides (glucose, xylose and arabinose), that can be used to generate biomass and valued chemicals. However, only a few studies have focused on *K. pneumoniae* for d-lactate production [12, 13]. The great potential for d-lactate by *K. pneumoniae* requires further development.

In our previous study [7], engineered *K. pneumoniae* was constructed by overexpressing the d-lactate dehydrogenase gene *ldhA* with knocking out the 1,3-PDO oxidordeuctase genes *dhaT* and *yqhD*. The resulting strain produced an extraordinary amount of d-lactate from glycerol under microaerobic conditions in fed-batch fermentation, which was much higher than that of the wild-type strain. And the optical purity is almost 100%, indicating high enzyme specificity of d-lactate dehydrogenase of *K. pneumoniae*. However, a significant amount of 1,3-PDO was still detected in the broth generated from unclear biosynthesis pathway, which complicated the purification process and decreased the yield of d-lactate.

*Klebsiella pneumoniae* can also utilize monosaccharides with 2,3-butanediol, d-lactate, ethanol, and acetate as main metabolites. It is speculated that a high yield of d-lactate could also be obtained from glucose with inhibiting the synthesis of by-products. In this study, the effects of single-gene and multiple-gene deletions in *K. pneumoniae* of acetate kinase (*ackA*), alcohol dehydrogenase (*adhE*), and acetolactate synthase (*budB*) were tested for their effects on d-lactate production. The effects of aeration on d-lactate production, metabolic flux and by-products, such as 2,3-butanediol, acetate, ethanol, and succinate, were also investigated. Under 2.5 vvm aeration condition, the triple gene-deficient strain produced 125.1 g/L d-lactate in 36 h with a yield of 0.91 g/g glucose.

## Methods

### Plasmids, strains, and the construction of plasmids

The plasmids, strains, and primers used in this study are listed in Table 1. *Klebsiella pneumoniae* was purchased from American Type Culture Collection (ATCC25955) and used as the parent strain for d-lactate production. *Escherichia coli* χ7213 was used for suicide vector preparation. The genomic DNA of *K. pneumoniae* was extracted using the E.Z.N.A.^®^ Bacterial DNA Kit (Omega Bio-tek, Georgia, America).

**Table 1:** Bacterial strains, plasmids, and primers used in this study

Strain, plasmid and primers	Description	Source
Strains		
*E. coli* DH5α	Cloning host	Lab collection
*E. coli* χ7213	Host strain for pRE112, DAP auxotrophic strain	[14]
Q1188	*K. pneumoniae* ATCC25955	ATCC
Q2657	*K. pneumoniae* ATCC25955 Δ*adhE*	This study
Q2666	*K. pneumoniae* ATCC25955 Δ*ackA*	This study
Q2699	*K. pneumoniae* ATCC25955 Δ*budB*	This study
Q2702	*K. pneumoniae* ATCC25955 Δ*budB* Δ*ackA* Δ*adhE*	This study
Q2710	*K. pneumoniae* ATCC25955 Δ*budB* Δ*adhE*	This study
Q2743	*K. pneumoniae* ATCC25955 Δ*budB* Δ*ackA*	This study
Plasmids		
pRE112	Suicide vector, R6k origin, chloramphenicol resistant	[15]
pRE112-Δ*budB*	Suicide vector for construction of Δ*budB* mutant	This study
pRE112-Δ*adhE*	Suicide vector for construction of Δ*adhE* mutant	This study
pRE112-Δ*ackA*	Suicide vector for construction of Δ*ackA* mutant	This study

Primers	Sequence (5′–3′)	Restriction enzymes
ID-pRE112	CAAGGCGACAAGGTGCTGATG	
Δ*adhE* construction		
1381	GCTCTAGAATGGCTGTTACTAATATCGC	*Xba*I
1382	CGACGCCGATAGCAGGTTTAC	
1383	GTAAACCTGCTATCGGCGTCGGTGCGTTCGGTGGTCTGGAT	
1384	GGGGTACCTCAGCCTTTACCGGAGCAAC	*Kpn*I
1385	ATAATGTCGAATCGAGCGAC	
1386	GCTTGTCGCGATGCTATCGC	
Δ*ackA* construction		
1387	GCTCTAGAATGTCGAGTAAGTTAGTAC	*Xba*I
1388	GCGTAGAGATAGGATTCTTC	
1389	GAAGAATCCTATCTCTACGCGAAGGCCTGGTGATGGGTAC	
1390	CGAGCTCTTATGCGGTCAGACGGCTGGC	*Sac*I
1391	ATCCTGCGCTACGCTAATGAC	
1392	CCTGCAGCTCGAATTATTGC	
Δ*budB* construction		
1448	GCTCTAGAGTCAGTCAGCTGGAAGCTC	*Xba*I
1449	ATAAGCTTCGCCACCTGGTC	
1450	GACCAGGTGGCGAAGCTTATCTGCGCATCGTTCGCGCCAT	
1451	CGAGCTCTTATCGCGATAATCTACCG	*Sac*I
1452	ATGGACAAACAGTATCCG	
1453	ACAGAATCTGACTCAGATG	
RT-qPCR		
16S rRNA_F	AAGCGTTAATCGGAATTAC	
16S rRNA_R	GCTACACCTGGAATTCTA	
16S rRNA_Probe	(FAM)CTCTACAAGACTCTAGCCTGCCAG(Eclipse)	
ldhA_F	GAAGCGGTATGTATCTTC	
ldhA_R	CAGGGCGATATATTTCAC	
ldhA_Probe	(FAM)CTTCAGCTCTTCCAGCACCG(ECLIPSE)	

All gene fragments were amplified by PCR using the genomic DNA of *K. pneumoniae* as a template. For *budB* (Genebank ID: 11848061) deletion, approximately 500-bp fragments upstream and downstream of this gene were amplified using the up-primers (1448 and 1449) and down-primers (1450 and 1451), respectively. The PCR mixture consisted of 1 ng of genomic DNA, 0.2 μmol of primers, 25 μL of double-distilled water, and 25 μL of PrimeSTAR MAX DNA Polymerase (TaKaRa, Dalian, China). The PCR was carried out at 95 °C for 5 min, followed by 30 cycles of 95 °C for 30 s, 55 °C for 30 s, and 72 °C for 30 s, with a final extension step of 72 °C for 10 min. Following gel electrophoresis, the PCR products were purified using the E.Z.N.A.^®^ Gel Extraction Kit. After obtained the two fragments, overlapping PCR was carried out to generate a ligated segment, which was cloned into the pRE112 suicide vector after digestion with the restriction enzymes *Xba*I and *Sac*I, resulting in the plasmid pRE112-Δ*budB*. The fragments of *ackA* (Genebank ID: 11848786) and *adhE* (Genebank ID: 11848216) were amplified by PCR using the corresponding primers (Table 1), and the same method was used to construct pRE112-Δ*ackA* and pRE112-Δ*adhE*. Δ*ackA* was cloned into pRE112 using the same sites, *Xba*I and *Sac*I. To generate pRE112-Δ*adhE*, the engineered segment was inserted into pRE112 with the restriction sites *Xba*I and *Kpn*I.

### Construction of the gene-deficient mutants

To construct gene-deficient mutants, *E. coli* strain χ7213, containing the plasmids pRE112-Δ*budB*, pRE112-Δ*ackA*, or pRE112-Δ*adhE*, was used as a donor in conjugation with *K. pneumoniae*. The mutation segments were introduced into *K. pneumoniae* by allelic exchange using the suicide vector pRE112, and double recombination was performed to obtain the mutants. For the first recombination, wild *K. pneumoniae* was incubated in Luria–Bertani (LB) media overnight with *E. coli* χ7213 (pRE112-Δ*budB*), then selected on an LB plate with 30 mg/L chloramphenicol before verification by PCR with the primers ID-pRE112 and 1384. The correct recombinant was incubated in LB media overnight for the second recombination, then cultured on 10% sucrose plates for selection and verified by PCR with the primers 1385 and 1386. Genomic DNA was used as a positive control, and the *budB* mutant was named as Q2699.

The other single gene-deficient mutants, Q2657 and Q2666, were constructed using the method described above. To make the double gene mutants, *E. coli* strain χ7213, which contained another recombinant suicide vector, was used as the donor in conjugation with a single gene-deficient mutant and then verified with antibiotics, sucrose, and PCR with the appropriate primers. The same strategy was used to introduce the third recombinant suicide vector into the double gene mutants to obtain the triple mutant strain.

### Media and growth conditions

*Escherichia coli* χ7213 was grown at 37 °C in LB medium with diaminopimelic acid (DAP) (50 mg/mL), and 30 mg/L chloramphenicol was added for suicide vector maintenance. The fermentation medium used in this study contained the following components (per litre): glucose, 20 g; NH_4_Cl, 5.4 g; yeast extract, 3 g; KH_2_PO_4_, 2 g; K_2_HPO_4_, 1.6 g; citric acid, 0.42 g; MgSO_4_·7H_2_O, 0.2 g; and 1 mL of trace elements stock solution. The trace element solution was composed of Na_2_MoO_4_·2H_2_O (0.005 g/L), H_3_BO_3_ (0.062 g/L), CuCl_2_·2H_2_O (0.17 g/L), CoCl_2_·6H_2_O (0.476 g/L), ZnCl_2_ (0.684 g/L), MnCl_2_·4H_2_O (2 g/L), FeCl_3_·6H_2_O (5 g/L), and concentrated HCl (10 mL/L).

For shake flask cultivation, the strains were cultured in a 250-mL flask containing 100 mL of medium at 37 °C in an orbital incubator shaker at a speed of 180 rpm. The samples were withdrawn to determine the cell mass, glucose, d-lactate, and by-products. 25% NH_3_·H_2_O was added to adjust the pH every 12 h. Glucose was determined by a SBA-40D biosensor analyser (Institute of Biology, Shandong Academy of sciences, China), and an additional 20 g/L glucose was added when the initial carbon source was nearly exhausted. All shaking experiments were carried out in triplicate. For analysis of the utilization of different carbon sources, 20 g/L xylose, arabinose, or mixed carbon sources containing glucose, xylose, and arabinose (the ratio of 1:1:1) was used instead of glucose.

Fed-batch fermentation was carried out in a Biostat B plus MO5L fermenter (Sartorius Stedim Biotech GmbH, Germany) with a working volume of 3 L. 200 mL of the seed solution was inoculated into the bioreactor and performed at 37 °C, with an agitation speed of 400 rpm. The broth pH was automatically maintained at 7.0 with ammonia. Sterile air was sparged at 0.5, 1.0, 1.5, and 2.5 vvm for different aeration conditions. After the initial glucose was nearly exhausted, the fed-batch mode was commenced by feeding a solution containing 70% (wt/v) glucose. The residue of glucose was controlled between 2 and 10 g/L during fermentation. Samples were withdrawn at intervals to determine the glucose residue, cell mass and the concentrations of metabolites.

### Real-time quantitative PCR for *ldhA* transcriptional level analysis

Total RNA from wild-type and mutant strains was isolated using Bacteria RNA Kit (Omega). The quantity and purity of the RNAs were determined by optical density measurements at 260 and 280 nm, respectively. RNA was reverse transcribed using TransScrip^®^ One-step gDNA removal and cDNA synthesis SuperMix Kit (TransGen Biotech). For each qPCR, 1 μL of sample, 10 μL of Premix Ex Taq (Probe qPCR) (2×) (TaKaRa), 0.4 μL of each primer (from a 10 μM of working solution, the primers presented in Table 1), and 0.8 μL of probe were added and supplemented with water to a final volume of 20 μL. The real-time quantitative PCR (RT-qPCR) was run on a LightCycler 480 system (Roche Diagnostics) with 16S rRNA as an internal reference. The qPCR program run consisted of a first step at 95 °C for 30 s and afterwards 40 cycles alternating between 5 s at 95 °C and 30 s at 60 °C. The samples were quantified by comparative cycle threshold (*C*t) method for relative quantification of gene expression [16].

### Analytical methods

Biomass was monitored using a UV visible spectroscopy system (Varian Cary 50 Bio, US) at 650 nm. The measurements were converted to dry cell weight (DCW) based on one unit of OD_650_ being equivalent to 0.284 g DCW/L. The fermentation products of d-lactate, 2,3-BDO, acetate, ethanol, and succinate were detected by HPLC with a refractive index detector (RI-150, Thermo Spectra System, USA) and ion exchange column (Aminex^®^ HPX-87H, 7.8 × 300 mm, BioRad) at 60 °C using 5 mM H_2_SO_4_ as the mobile phase, with a flow rate of 0.5 mL/min. The optical purity of d-lactate was measured using the reported method [17]. The carbon distribution was calculated based on glucose consumption, and that contained in the main metabolites and biomass. The fraction for cell growth was determined with the elemental composition of C_4_H_7_O_2_N [18].

## Results and discussion

### Effect of gene deletion on cell growth

**Fig. 1:**
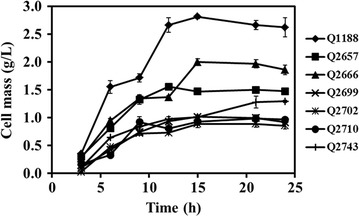
Growth curves of the parent and mutants of *K. pneumoniae* with glucose as the carbon source. The experiment was performed in a shaking flask in triplicate, and the standard deviation is shown

To investigate the effects of single-gene deletion or multi-gene deletions of *budB*, *ackA*, and *adhE* on cell growth, the gene-deficient mutants and wild-type strain were cultured under the same conditions in shake flask (Fig. 1). Obvious inhibition on cell growth was observed after the single gene was deleted. In particular, the biomass accumulation of the *budB* deletion mutant Q2699 was less than half that of the parent strain at 15 h. Growth of the multi-gene knockout strains Q2702, Q2710 and Q2743 was inhibited more severely. The DCW of the triple mutant strain Q2702 was only 0.89 g/L in the stationary phase. The results demonstrated that blocking the 2,3-BDO pathway significantly reduced cell growth, which is consistent with previous reports [19]. Due to NADH requirements for 2,3-BDO and ethanol formation in *K. pneumoniae*, blocking these by-product pathways could alter the intracellular redox balance, which may act as an inhibitor of cell growth.

### Metabolic profiles of single-gene mutants and their effect on d-lactate production

**Fig. 2:**
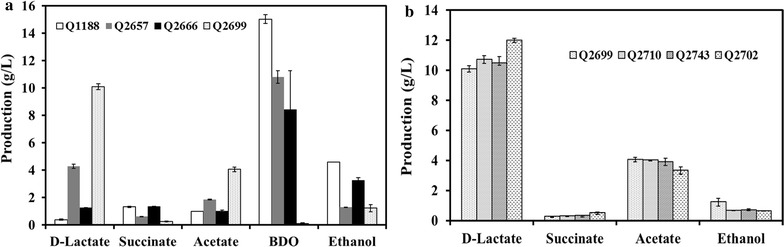
Metabolite profiles of the parent and mutants of *K. pneumoniae*. The experiment was performed in a shaking flask in triplicate, and the standard deviation is shown. **a** Metabolic profiles of single-gene mutants and their effect on d-lactate production. **b** Metabolic profiles of the multi-gene mutants and their effect on d-lactate production

The production of the main metabolites from glucose by single-gene mutants and wild-type strain was investigated (Fig. 2a). As shown in Fig. 2a, the three mutants had different metabolic performances compared to the wild-type strain. Inactivation of *ackA* clearly resulted in a reduction in 2,3-BDO and ethanol production. The production of acetate essentially remained the same because complex metabolic pathways of acetate in microbes. A previous report also demonstrated that deleting the primary acetate pathway genes *pta*-*ackA* of *K. pneumoniae* had no effect on the formation of acetate [20]. However, in addition to *ackA* and *pta*, there are still many other genes related to acetate secretion. The mutant *E. coli* with *ptsG*, *poxB*, *pta*, and *iclR* gene knocked out was confirmed to accumulate only 10% acetate of the wild strain [21]. It is also proved that acetate excretion is generally caused by an imbalance between the glycolytic pathway and tricarboxylic acid (TCA) cycle. Thus, controlling glucose uptake or enhancing the activity of the TCA cycle might decrease acetate accumulate. Our previous study has revealed that knockout of *arcA*, a global regulator which repress the TCA cycle, exhibited surprising efficacy in inhibiting acetate synthesis [22]. Theoretically, all the above methods can be used for reducing acetate secretion of *K. pneumoniae* in future studies. Although acetate accumulation was not inhibited, the *ackA* mutant strain produced 1.25 g/L d-lactate at 24 h compared to 0.38 g/L d-lactate of the wild-type strain.

d-Lactate production reached 4.29 g/L at 24 h for the Δ*adhE* strain Q2657, which was significantly higher than those of the wild-type strain Q1188 and Δ*ackA* strain Q2666. For by-products, Q2657 produced 1.29 g/L ethanol and 0.62 g/L succinate, which were 72 and 53% lower than those produced by the wild-type strain, respectively. However, acetate production was improved to 1.85 g/L. In glucose metabolism of *K. pneumoniae*, glucose is oxidized to pyruvate and then converted into acetyl-CoA; acetyl-CoA can be used for acetate and ethanol production in addition to the TCA cycle. It was speculated that more acetyl-CoA flowed into the acetate pathway after the ethanol pathway was restrained with the *adhE* deletion.

**Fig. 3:**
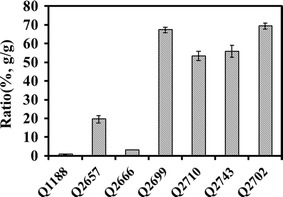
Conversion ratio of d-lactate production to consumed glucose by the *K. pneumoniae* parent strain and mutants. The experiment was performed in a shaking flask in triplicate, and the standard deviation is shown

Knockout of *budB* resulted in only 0.1 g/L 2,3-BDO production, which confirmed that *budB* was the major 2,3-BDO synthesis gene in *K. pneumoniae*. The largest production of d-lactate (10.1 g/L) was achieved by the Δ*budB* strain Q2699, and a yield of 67% was obtained at 24 h, while those of Q1188, Q2657, and Q2666 were only 0.76, 19.54, and 3.13%, respectively (Fig. 3). These results revealed that deletion of the 2,3-BDO pathway significantly triggered metabolic flux redistribution and generated excess d-lactate production. By contrast, 2,3-BDO production was successfully improved by a lactate dehydrogenase-deficient mutant [23]. Additionally, the recombinant strain produced less succinate and ethanol, while acetate production increased to 4.1 g/L.

### Metabolic profiles of the multi-gene mutants and their effect on d-lactate production

As the Δ*budB* strain Q2699 obtained the highest production and yield of d-lactate among the single gene mutants, double and triple mutant strains were constructed based on it. As shown in Fig. 2b, the double mutant strains (Q2710 and Q2743) produced slightly more d-lactate than Q2699, and the by-products production was further reduced. However, the d-lactate yields were 56 and 53%, which were lower than those of Q2699 (Fig. 3). The triple gene-deficient strain exhibited the highest d-lactate synthesis efficiency. Using the Δ*budB*Δ*ackA*Δ*adhE* strain Q2702, 11.99 g/L d-lactate was obtained, which was 19% higher than that of Q2699, with a yield of 69%. The production of acetate and ethanol resulted in reductions of 18 and 49% compared with those of Q2699, respectively. With *budB* deletion, 2,3-BDO was not detected in all of the multi-gene mutants, and the production of succinate remained at a low level.

Formation of the main by-products 2,3-BDO, ethanol, and succinate require NADH, which compete with the d-lactate pathway [24]. More NADH can be used to produce d-lactate after blocking the primary by-products pathway by deleting *budB*, *ackA*, and *adhE*. In addition, the transformation of glucose to each mol of 2,3-BDO generates 2 mol of CO_2_, which means that more carbon flows into products with blockage of 2,3-BDO synthesis. The routes outlined above may be the primary causes of the higher production and yield of d-lactate achieved by the mutant strains.

### Effect of deleting the by-products synthesis genes on the *ldhA* transcriptional level

**Fig. 4:**
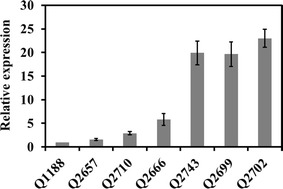
Relative expression level of *ldhA* in *K. pneumoniae* parent strain and mutants by RT-qPCR. 16S rRNA was used as an internal reference

Mutant strains generated increased d-lactate production, and the underlying causes need to be revealed. The total RNAs of the wild-type and mutant strains were extracted, and all of the cDNA samples were diluted to the same concentration for RT-qPCR. As shown in Fig. 4, the transcriptional levels of the lactate dehydrogenase gene *ldhA* were higher than those of the wild-type strain in all of the mutant strains, especially Q2699, Q2702 and Q2743 (19.7, 23.0, and 19.9 times of wild-type strain respectively). The results suggested that the deletion of the by-products synthesis genes up-regulated the transcriptional levels of *ldhA*, which possibly explains the higher d-lactate production by the mutant strains. In previous studies, the specific activity of LDH drastically increased after blocking the 2,3-BDO pathway [25] or with the combined deletion of *adhE* and *ackA*-*pta* [26], which could result in higher d-lactate production. Additionally, blocking the 2,3-BDO pathway affected the metabolic network of *K. pneumoniae* [24]: 511 genes were differentially regulated in the *budA* deletion mutant compared to the wild type. Many genes related to energy production and coenzyme transport were up-regulated, which may enhance metabolite formation.

### Potentiality analysis for carbon source utilization by Q2702

*Klebsiella pneumoniae* can use a wide spectrum of carbon sources. In addition to glucose, d-xylose and l-arabinose were also tested with Q2702 in this study. As shown in Table 2, each individual sugar and the mixtures were able to be effectively used by the mutant strain Q2702 and generated a similar biomass in flask fermentation. However, the production and yield of d-lactate were significantly decreased when xylose, arabinose, or mixtures of carbon sources were used. For the primary by-products, acetate production decreased and ethanol accumulation enhanced slightly when xylose was used, and succinate production improved when arabinose was used.

**Table 2:** Metabolic profiles of Q2702 using several carbon sources in 24-h-flask cultivation

Carbon source	d-Lactate (g/L)	Succinate (g/L)	Acetate (g/L)	Ethanol (g/L)	Cell mass (g/L)	Consumed carbon (g/L)	d-Lactate yield (g/g)
Glucose	11.99 ± 0.14	0.48 ± 0.08	3.33 ± 0.24	0.62 ± 0.01	1.13 ± 0.09	17.31 ± 0.22	0.69
d-Xylose	9.34 ± 0.21	0.43 ± 0.02	2.67 ± 0.14	0.73 ± 0.13	0.95 ± 0.11	15.81 ± 0.25	0.59
l-Arabinose	8.87 ± 0.41	0.91 ± 0.16	3.25 ± 0.16	0.51 ± 0.07	1.07 ± 0.10	16.19 ± 0.42	0.55
Mixture	8.36 ± 0.37	0.41 ± 0.04	3.11 ± 0.09	0.57 ± 0.05	1.08 ± 0.08	15.57 ± 0.16	0.54

In addition, the strain demonstrated a preference for glucose in mixtures of carbon; glucose was exhausted, while a large amount of xylose and arabinose remained at the end of fermentation. Based on the amount of carbon consumed, xylose utilization was slower than arabinose and glucose. *Klebsiella oxytoca* was also evaluated for its ability to ferment mixtures of the sugars l-arabinose, d-xylose and d-glucose (1:1:1); approximately 47% of xylose was unutilized after 114 h fermentation, while glucose was exhausted at 24 h and arabinose was exhausted at 96 h [27]. As glucose, xylose, and arabinose are the primary components of lignocellulose hydrolysates [28], it is presumed that *K. pneumoniae* has great potential to ferment lignocellulosic hydrolysates into valued products.

### d-Lactate production and metabolic profiles of Q2702 in fed-batch fermentation

**Fig. 5:**
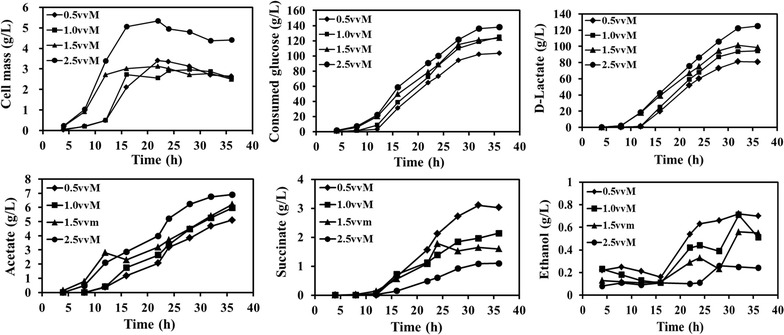
Cell mass, consumed glucose and primary metabolites in fed-batch fermentation at different aeration rates in a 5-L bioreactor

As the triple mutant strain Q2702 exhibited the highest capacity for producing d-lactate, fed-batch fermentations were carried out to further enhance production. To investigate the effect of aeration on the production of d-lactate, fed-batch fermentations were conducted at different aeration rates of 0.5, 1.0, 1.5 and 2.5 vvm (Fig. 5). With enhanced aeration, the growth rate and cell mass were improved dramatically at 2.5 vvm, which agreed with a previous report [7, 17]. The maximum cell mass achieved was 5.34 g/L at 22 h. The biomass decreased slightly after 24 h of fermentation, possibly due to cellular toxicity caused by the high lactate concentration.

**Fig. 6:**
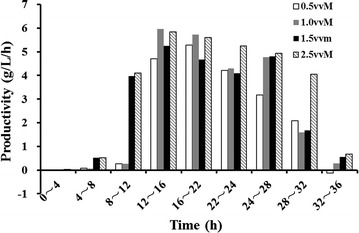
Productivity of d-lactate at different stages of fed-batch fermentation at different aeration rates

The aeration conditions significantly impacted d-lactate production. The production was only 80.89 g/L at 36 h, with a yield of 0.78 g/g glucose under 0.5 vvm. With increasing aeration rates, d-lactate production improved to 94.6 and 98.4 g/L under 1.0 and 1.5 vvm, respectively, and the maximum concentration of d-lactate of 125.1 g/L was reached under 2.5 vvm, with a yield of 0.91 g/g glucose. The accumulation of d-lactate followed a similar trend with cell growth. Cells grew slowly due to the lag phase during the first few hours, and little d-lactate was produced without sufficient cells. During the apparent logarithmic phase, production accumulated rapidly, and productivity was higher than 5 g/L/h (Fig. 6). The highest productivity of 5.96 g/L/h was achieved at 12–16 h under 1.0 vvm. However, the average productivity of 5 g/L/h lasted for as long as 24 h at 2.5 vvm, and 5.85 g/L/h was obtained from 12–16 h, which was very close to the highest productivity under 1.0 vmm. The productivity was severely reduced as cells declined and died during the late stationary phase during all of the fed-batch fermentations.

Table 3 summarizes the d-lactate fermentation performances from different carbon sources by different strains. Glucose and glycerol were the main carbon sources used for d-lactate fermentation. Some inexpensive carbon sources, such as sweet potato, corn starch, and monosaccharides from cellulosic hydrolysates, have been used in recent years. *Lactobacillus* was the most commonly used genus for d-lactate production, and *L. coryniformis* produced 186.4 g/L d-lactate after 60 h of fermentation from sweet potato with an average productivity of 3.1 g/L/h [29]. However, *Lactobacilli* usually have two or more lactic dehydrogenases and prefer to racemic lactic acid during fermentation [36, 37]. *Escherichia coli* has also been genetically modified to produce d-lactate; more than 120 g/L was obtained in previous paper [5]. Both *Lactobacilli* and *Escherichia* needed nitrogen or carbon dioxide sparging to maintain anaerobic environment for lactate accumulation, which would increase the production costs. There are few studies on d-lactate formation by *K. pneumoniae* with glycerol as the carbon source [7, 12, 13]. To our knowledge, no report regarding d-lactate fermentation from glucose by *K. pneumoniae* has been published. The high productivity, yield, and optical purity obtained in this study demonstrated that *K. pneumoniae* is an excellent producer of d-lactate from glucose.

**Table 3:** Comparison of d-lactate production by different strains using different carbon sources

Carbon source	Organism	Relevant genotype	d-Lactate production (g/L)	Yield (g/g)	Productivity (g/L/h)	Optical purity (%)	References
Glucose	*K. pneumoniae*	Δ*budB* Δ*ackA* Δ*adhE*	125.1	0.91	3.48	99.9	This study
Glucose	*E. coli*	Δ*ack* Δ*pps* Δ*frdA* Δ*pflB* Δ*dld* Δ*poxB* Δ*adhE*, *ldhA*	122.8	0.84	4.32	NA	[5]
Glucose	*Sporolactobacillus inulinus*	*pyk*	115.1	0.81	0.68	NA	[30]
Glucose	*Corynebacterium glutamicun*	Δ*ldhL*, *ldhA*	120	0.87	4	99.9	[31]
Glucose	*Saccharomyces cerevisiae*	Δ*pdC1*, *ldhA*	61.5	0.61	0.85	99.9	[6]
Glycerol	*K. pneumoniae*	Δ*dhaT* Δ*yqhD*, *ldhA*	142.1	0.82	2.96	~ 100	[7]
Glycerol	*K. pneumoniae*	Δ*budC*, *ldhA*	68.4	0.78	1.22	NA	[12]
Glycerol	*K. pneumoniae*	–	59	0.47	1.13	NA	[13]
Glycerol	*E. coli*	Δ*pta* Δ*adhE* Δ*frdA* Δ*dld* pZS*glpKglpD*	32	0.80	0.54	99.9	[32]
Glycerol	*E. coli*	Δ*pta*-*ack* Δ*pps* Δ*pflB* Δ*dld* Δ*poxB* Δ*adhE* Δ*frdA*, *ldhA*	100.3	0.75	2.78	99.97	[33]
Sucrose	*E. coli*	Δ*adhE* Δ*frdABCD* Δ*pta* Δ*pflB* Δ*aldA* Δ*cscR*	85	0.85	1	98.3	[34]
Sweet potato	*Lactobacillus coryniformis*	–	186.4	0.85	3.11	NA	[29]
Corn starch	*L. plantarum*	Δ*ldhL*::*amyA*	73.2	0.73	1.53	99.6	[35]
Glucose, xylose, arabinose	*L. plantarum*	Δ*ldhL* Δ*xpK1* Δ*xpK2*	74.2	0.78	2.06	99.5	[8]

As an opportunistic pathogen, *K. pneumoniae* could pose security risks and limit its industrial applications. The virulence factors of *K. pneumoniae* such as capsule, fimbriae, lipopolysaccharide, adhesins, and siderophores have been identified [38]. And a number of virulence genes which contribute to bacterial pathogenesis such as *wabG*, *fimA*, *magA*, *cps*, have been systematically analyzed and verified in *K. pneumoniae* [39]. Genetic modification is worth trying to eliminate the pathogenic characteristics. Some single gene-deficient mutants have generated in previous studies, the pathogenicity of the mutant strains was reduced dramatically, the growth and the desired product yield were unaffected [40, 41]. What calls special attention is that some new non-pathogenic *Klebsiella* strains were isolated [42, 43], which will enhance the competitive edge in industrial applications. Some special methods such as cell encapsulation and solar photocatalysis were also evaluated for their potential to diminish the risk of *K. pneumoniae* [44, 45].

The removal of *K. pneumoniae* cells is another obstacle to its commercial applications. *K. pneumoniae* cells are difficult to separate from fermentation broth because of its capsule and fimbriae, which complicated the downstream processing [46]. Mutant strains devoid of capsule, fimbriae, and lipopolysaccharide could facilitate cell removal by high-speed centrifugation. As a traditional method, centrifugation can remove most of the macromolecules, but the high investment and energy consumption make it to be an unsatisfactory method. Membrane filtration and flocculation are the other two most commonly used methods to remove the cells from fermentation liquors. Although the progress of membrane separation technology has greatly improved its efficiency recently, the membrane fouling and poor material properties still need to be resolved [47]. Flocculation is an important method for the liquid–solid separation process in a number of industrial applications, which have attracted much attention due to its simplicity, and applied to precipitation of different microbial cells in biological industries. Flocculation precipitation is predicted as the most promising method in industrial scale if cheap and effective flocculants are available, some flocculants such as chitosan and polyacrylamide have already been tested for this purpose [48].

The profiles of the by-products are also shown in Fig. 5. The major by-product was acetate, and accumulation was enhanced with increasing oxygen availability. Here, 6.9 g/L acetate accumulated under 2.5 vvm, while 5.11 g/L was obtained at 0.5 vvm. Succinate and ethanol were also detected under 0.5 vvm at concentrations of 3.03 and 0.7 g/L, respectively. With increasing oxygen availability, the production of these two by-products decreased; only 1.1 g/L succinate and 0.24 g/L ethanol were accumulated at 2.5 vvm.

### Carbon distribution under different aeration rates

To further understand the metabolism of the mutant strain, the carbon distribution was determined under different aeration rates. As shown in Table 4, the biomass formation increased with aeration: it was 1.67 times higher at 2.5 vvm than that at 0.5 vvm. Under higher aeration rates, more d-lactate and biomass generation were the primary reasons for the high carbon recovery. The transformation of glucose to each mol d-lactate generates 1 mol of NADH; more d-lactate production resulted in greater NADH formation, and the reducing power required by cell metabolism from oxidative pentose phosphate was reduced; subsequently, CO_2_ emission from the PP pathway was reduced, which improved carbon recovery. Here, 90.6% carbon was distributed into d-lactate, and a maximum carbon recovery of 100.2% was achieved at 2.5 vvm. As reported, the organism took up oxygen at a higher rate and converted more glucose to CO_2_ with decreased oxygen availability [49], which led to greater carbon losses. The total carbon recovery was < 90%, and only approximately 75% of carbon was used for d-lactate production at 0.5, 1.0, and 1.5 vvm. The actual carbon recovery should be lower than calculated in this study, as the glucose requirements for cell growth were reduced with the addition of yeast extract to the medium.

**Table 4:** Carbon balance in fed-batch fermentations under different conditions

Substrates or metabolites	Aeration rate			
	0.5 vvm (mmol)	1.0 vvm (mmol)	1.5 vvm (mmol)	2.5 vvm (mmol)
Glucose	3443.3	4163.3	4113.3	4600.0
Biomass	96.9	93.8	91.7	161.5
d-Lactate	2696.3	3153.3	3280.0	4170.0
Acetate	170.3	198.7	206.7	230.0
Succinate	102.7	72.5	54.2	37.3
Ethanol	30.4	22.2	23.9	10.4
Lactate recovery (%)	78.3	75.7	79.7	90.6
Carbon recovery (%)	89.9	85.1	88.9	100.2

## Conclusions

The effects of single-gene and multiple-gene deletions in *K. pneumoniae* on d-lactate production from glucose via *budB*, *ackA*, and *adhE* were tested. The triple mutant had the highest capacity for producing d-lactate. The aeration rate played a key role in d-lactate accumulation, and 125.1 g/L of d-lactate with a yield of 0.91 g/g glucose in 36 h was produced by the triple mutant at 2.5 vvm. This study demonstrated that *K. pneumoniae* is an excellent producer of d-lactate from glucose and also showed the feasibility of producing d-lactate from pentose sugars, such as xylose and arabinose.
